# Method to Predict Salt Expansion Deformation in Cement-Stabilized Macadam Under Sulfate Attack Based on Pore Evolution

**DOI:** 10.3390/ma18214863

**Published:** 2025-10-23

**Authors:** Xiangyu Li, Xuesong Mao, Pei He, Qian Wu

**Affiliations:** School of Highway, Chang’an University, Xi’an 710064, China; 2021021012@chd.edu.cn (X.L.); 2022221274@chd.edu.cn (P.H.); wuqian@chd.edu.cn (Q.W.)

**Keywords:** cement-stabilized macadam, pore characteristics, sulfate attack, CT scanning, expansion deformation

## Abstract

Cement-stabilized macadam often shows salt expansion deformation under the action of a sulfate attack, and its pore structure determines its ability to accommodate this deformation. In this paper, the influence of the pore structure of cement-stabilized macadam on its macroscopic deformation is analyzed using a single-grain salt expansion deformation test, scanning electron microscopy (SEM), and computerized tomography (CT) scanning. The results show that ettringite and sodium sulfate decahydrate crystals are key factors in salt expansion deformation. In addition, we find that when the sulfate content increases from 0% to 5%, the porosity of the mixture decreases by 1.5%, the proportion of primary pores increases by 12.1%, and the linear expansion rate increases by 0.05%. Finally, a salt expansion deformation prediction model for cement-stabilized macadam is proposed, which takes the porosity of the mixture, the proportion of graded pores, and the deformation influence factor as parameters, and the error is found to be less than 10%.

## 1. Introduction

In some areas of Inner Mongolia and Xinjiang in China, the road base frequently shows signs of arch cracking [1,2,3]. Many scholars have carried out systematic research by means of laboratory tests, theoretical analysis, and numerical simulation [4,5,6,7,8,9,10,11,12,13,14,15,16]. Several studies have suggested that the main cause is sulfate attack on cement-stabilized macadam, and expansion products such as ettringite and sodium sulfate decahydrate are generated in the mesopores of the structure [17,18,19,20]. These expansion products absorb a high number of water molecules to expand by 2–3 times, which leads to volume expansion and cracking [21,22]. Therefore, pore evolution is closely related to macroscopic deformation in cement-stabilized macadam under sulfate attack.

The existing research on sulfate attack in cement-based materials has mainly focused on cement concrete [23,24,25,26,27,28,29,30,31]. Wang et al. [32] found that sulfate attack products tended to be generated in small pores and that the overall porosity of the mixture was significantly reduced. Wu et al. [33] found that in the erosion process, the composite expansion of ettringite led to cracks in the microstructure of the mixture, and with the aggravation of the erosion reaction, the number of cracks increased. Siad et al. [34] studied changes in the microstructure of cement concrete under sulfate attack and found that sulfate attack caused microcracks and damage in the structure. They also observed that several crystals, such as ettringite, were formed in the pores during the erosion reaction, which led to the expansion and deformation of the mixture.

In summary, scholars have highlighted the important role of mesopores during sulfate attack in cement concrete. However, the impact of different pore sizes has rarely been considered in the literature, and methods to accurately predict salt expansion deformation are still lacking. Generally speaking, the pore structure of the specimen directly determines its ability to accommodate salt expansion, and the effects of different sizes of pores on deformation are expected to be different.

In this paper, a salt expansion test will be carried out on single-particle cement-stabilized macadam. Due to the limitation of the particle range, the pore structure size formed in the mixture will be relatively singular. Therefore, by testing the volume deformation of the sample with this structure, we will determine the impact factor of graded pores in salt expansion. At the same time, computerized tomography (CT) scanning will be used to determine the porosity and pore distribution of the specimen with the target gradation, and the evolution law of the mesopores of the material under sulfate attack will be analyzed. Based on our experiments, the impact factor of graded pores in deformation caused by salt expansion and the volume ratio of graded pores will be obtained. By calculating the expansion deformation caused by pores at all levels and summarizing them, we will develop a prediction model for salt expansion deformation in cement-stabilized macadam under the target gradation. The model will consider the influence of different pore sizes and reveal the mechanism behind salt expansion deformation from the perspective of mesopores, which will provide a theoretical reference for further study in this field.

## 2. Test Scheme

In order to predict salt expansion deformation in cement-stabilized macadam under sulfate attack, it is necessary to determine the impact factors and volume proportion of each pore. Therefore, we designed a single-particle cement-stabilized macadam salt expansion test, as well as a salt expansion test under the target gradation. The former simplified the pore size of the mixture by limiting the particle size and allowed us to analyze the impact factors for different pores. The latter was combined with CT scanning technology to reconstruct the three-dimensional pore structure of the specimen and allowed us to analyze the evolution law and volume ratio of mesopores under sulfate erosion.

### 2.1. Salt Expansion Deformation Test in Single-Particle-Size Cement-Stabilized Macadam

In order to study the influence of the mesopore size of the structure on salt expansion deformation, in this paper, a single-sieve-size macadam was used to form a mixture with cement and anhydrous sodium sulfate, and a structure with a relatively singular mesoscopic pore size was constructed. Based on this, salt expansion deformation tests were carried out in cement-stabilized macadam samples with different sulfate contents and pore sizes.

#### 2.1.1. Single-Particle-Size Cement-Stabilized Macadam Salt Expansion Deformation Test Program

According to the requirements regarding the key sieve size for the skeleton-dense gradation in the current specification [35], particle sizes of 0.075 mm, 0.3 mm, 0.6 mm, 1.18 mm, 2.36 mm, and 4.75 mm were selected for the experiments. During the experiments, we found that when the macadam size was greater than 4.75 mm, the mixture lacked fine materials, which made it difficult to form a cemented part under the same cement dosage, and the specimen did not produce obvious deformation within the salt content range used. Therefore, only the macadam with a size below 4.75 mm was selected for the tests. Anhydrous sodium sulfate was used as the test salt, and the sulfate contents used were 0, 1.5%, 2.5%, 3.5%, and 5% of the gravel mass. The cement content was always 4%. The specific test scheme is shown in Table 1.

#### 2.1.2. Theoretical Aperture Analysis

**Figure 1:**
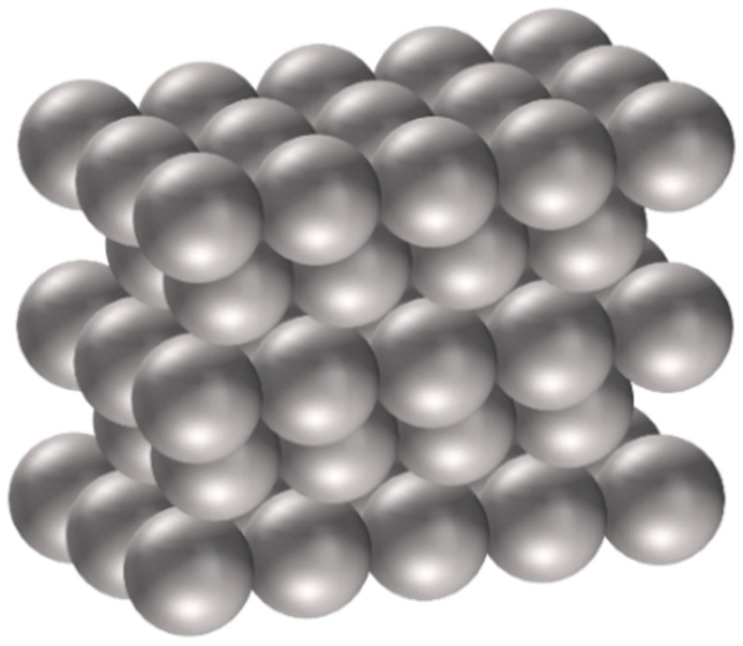
The accumulation mode of particles.

In our experiment, macadam with a single particle size was used, and the accumulation mode of the particles was similar to that of the atoms in the crystal cell, as shown in Figure 1. The closest packing of spheres of the same size can take two basic forms: hexagonal packing (HCP) and face-centered cubic packing (FCC). The space utilization rate of both is 74.05%. According to the stage filling theory, the pore diameter formed by single-size particles is 0.72 times the diameter of the particles used [36]. The theoretical pore size was calculated as shown in Table 1.

In order to facilitate the quantitative analysis of pores, different pores are classified in this paper. The equivalent sphere volume is used to represent the pore volume, and the specific model pore classification is shown in Table 2.

#### 2.1.3. Sample Preparation and Deformation Test

**Figure 2:**
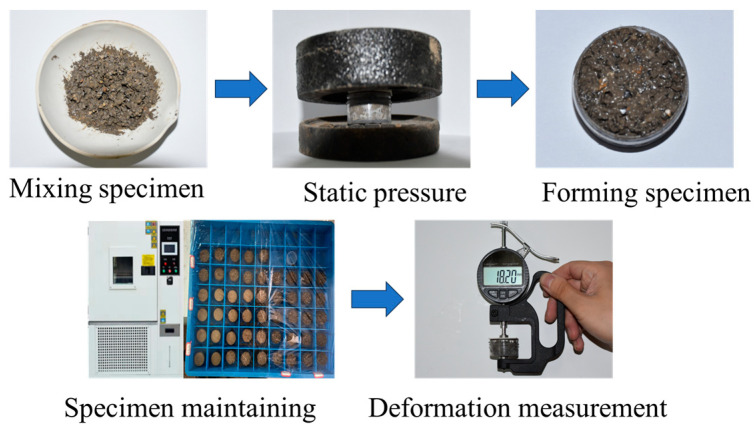
Specimen preparation and test process.

As shown in Figure 2, for the deformation test, we adopted a small cylindrical specimen with a size of 30 mm in diameter × 15 mm in height, and the specimen box satisfied the size effect of the maximum particle size. Each specimen was formed via static pressure, and the degree of compaction was 98%. After forming, specimens were placed in an environmental box for 28 days with a the control temperature of 25 ± 2 °C, and then, the expansion deformation was measured. Each parallel group included 3 specimens. The expansion deformation was measured using a digital micrometer with an accuracy of 0.01 mm, and the average value was calculated.

### 2.2. Target Gradation Cement-Stabilized Macadam Salt Expansion Deformation Test

A large, cylindrical specimen was used in the test, with a size of 150 mm in diameter × 150 mm in height. Each sample was formed via static pressure with the universal press, and the degree of compaction was 98%. The specimens were also placed in an environmental box of 25 ± 2 °C for 28 days after molding, and then, the expansion deformation was measured. The main factor affecting the deformation of the specimen under the target gradation was the sulfate content, so specimens with sulfate contents accounting for 0%, 2.5%, and 5% of the fine-particle mass (below 1 mm) were developed. The amount of cement was 4%, and the target gradation is shown in Table 3. Each parallel group had 3 specimens. The expansion deformation was measured using the vernier caliper method, and the average value was obtained.

### 2.3. CT Scanning Technology and Image Preprocessing

**Figure 3:**
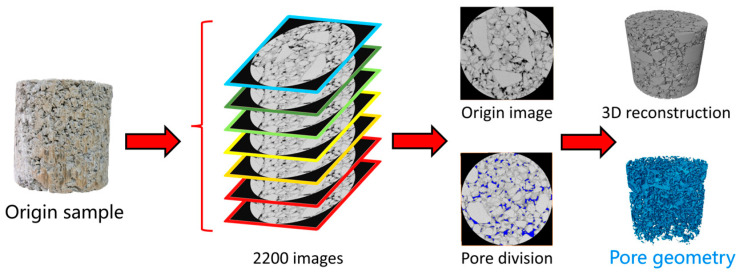
CT scanning equipment and process.

**Figure 4:**
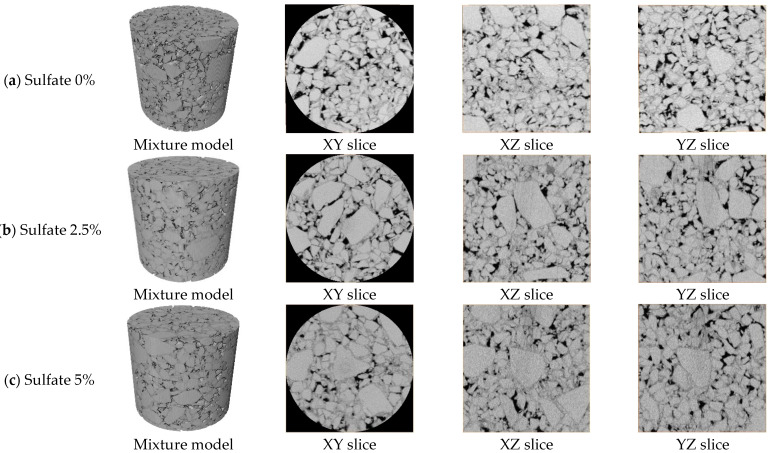
Scan images with different sodium sulfate contents.

In order to ensure the test accuracy of CT scanning, industrial CT was selected for scanning. The machine model was the YXLON CT Compact fan-beam CT (Hamburg, Germany) with a voltage of 450 kV, a resolution of 66 μm, and a scanning interval of 66 μm, as shown in Figure 3. The scanning results were processed using Avizo 3D 2022.2 software, and the scanning section of a typical sample after pretreatment is shown in Figure 4.

## 3. Test Results and Analysis

### 3.1. Deformation Analysis in Single-Particle Cement-Stabilized Macadam

**Figure 5:**
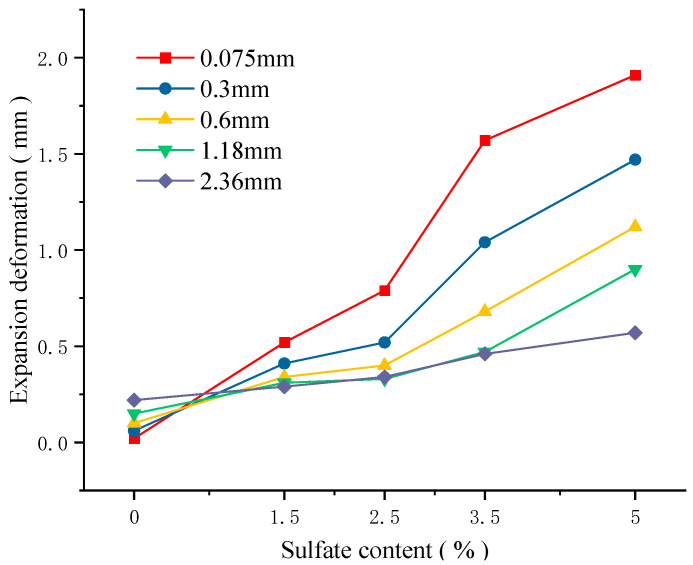
Expansion deformation in single-particle cement-stabilized macadam.

Figure 5 shows the expansion deformation in single-particle-size cement-stabilized macadam after 28 days of molding with different sulfate contents. It can be seen that, for all particle sizes, the expansion deformation in the sample increases with the increase in sulfate content. This shows that the sulfate content affects the formation of the expansive crystalline phase. When the sulfate content in the sample increases, the yield of crystals such as ettringite and sodium sulfate decahydrate during erosion also increases. The crystals generated in the meso-structure cause a sharp increase in internal expansion stress and ultimately lead to a significant increase in expansion deformation in the sample. In Figure 5, the levels of expansion deformation with different particle sizes are compared and analyzed. It can be seen that the expansion deformation in the sample is inversely proportional to the particle size of the macadam. The smaller the particle size, the smaller the mesopore size of the mixture structure and the greater the expansion deformation of the specimen under the same sulfate content. For example, when the sulfate content is 5%, the expansion deformation in the mixture with a gravel particle size of 0.075 mm is increased by 1.34 mm (+235%) compared with the mixture with a gravel particle size of 2.36 mm. According to the analysis of the expansion mechanism of sulfate erosion, when the sulfate content is constant, the crystal phase generated in the mixture can be determined. Therefore, the larger the initial porosity inside the structure, the stronger the ability to accommodate the crystalline phase, and the expansion deformation of the sample under salt erosion is relatively small. In addition, when the mixture does not contain salt, the specimen shows little expansion deformation. This is mainly because, under the condition of artificial maintenance, different parts of the sample undergo different degrees of dry–wet cycles, resulting in the loosening of the sample. Specifically, the larger the particle size of the mixture, the more loose deformation there is in the sample.

Through the previous analysis, the sample will produce a little loose deformation due to the partial dry–wet cycle, which will affect the total deformation of the sample. Therefore, the expansion deformation of the sample without salt is taken as the correction amount, and the expansion deformation in the sample is divided into loose deformation and salt expansion deformation. The modified formula for salt expansion deformation is given as Equation (1):

(1) $$\Delta L_{W}=\Delta L-\Delta L_{0}$$

where

$\Delta L_{W}$ is the salt expansion deformation of the specimen when the salt content is *w*, mm;
ΔL is the total deformation of the specimen, mm;
$\Delta L_{0}$ is the deformation of the specimen when the salt content is 0, mm.

**Figure 6:**
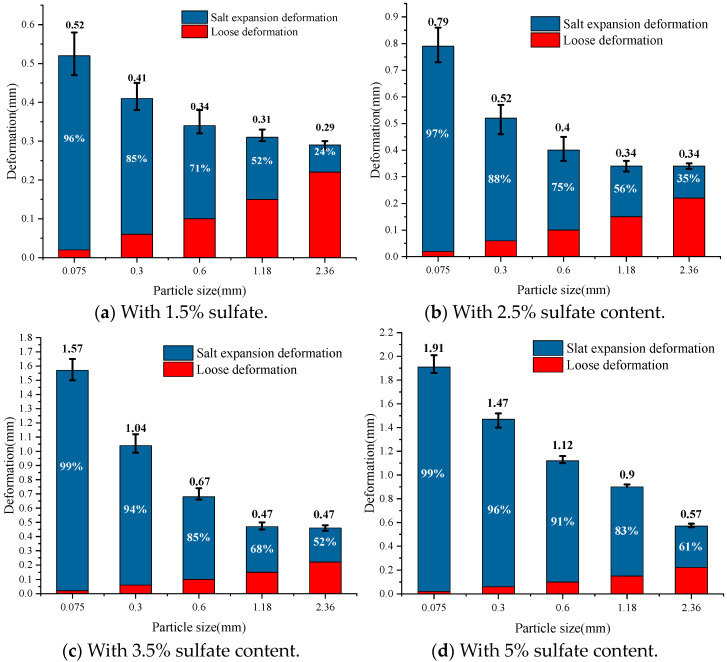
Salt expansion and loose deformation in samples with different particle sizes.

The deformation law for cement-stabilized macadam with different particle sizes is further analyzed, as shown in Figure 6. The actual salt expansion deformation caused by sulfate can be determined after the introduction of loose deformation for correction. Under the same salt content, the salt expansion deformation of the specimen decreases with the increase in the particle size of the macadam. The loose deformation increases with the increase in particle size. With the increase in sulfate content, the proportion of salt expansion deformation to the total deformation increases significantly. Specifically, when the sulfate content is 1.5%, the total deformation of the mixture with 0.075 mm particle size is 0.52 mm, and salt expansion deformation accounts for 96% of the total deformation. The total deformation of the mixture with a particle size of 2.36 mm is 0.29 mm, and the salt expansion deformation accounts for 24% of the total deformation. When the sulfate content is increased to 5%, the total deformation of the mixture with a 0.075 mm particle size is 1.89 mm, and the salt expansion deformation accounts for 99% of the total deformation. The total salt deformation of the mixture with a 2.36 mm particle size is 0.35 mm, and salt expansion accounts for 61% of the total deformation. It can be seen that the mixture with a smaller particle size is obviously affected by the sulfate content; the overall expansion deformation is larger, as is the proportion of salt expansion deformation. The mixture with a larger particle size is more stable under the influence of sulfate, the overall expansion deformation is smaller, and the proportion of salt expansion deformation is smaller.

### 3.2. Deformation Mechanism

**Figure 7:**
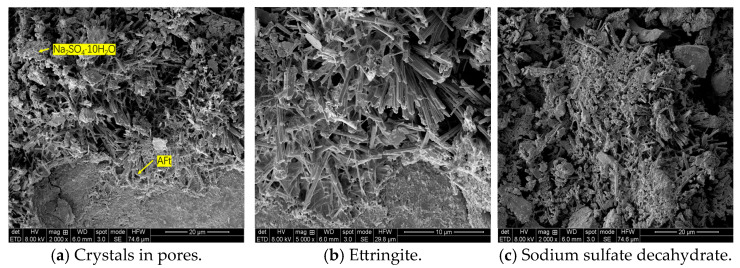
SEM images of the sample.

**Figure 8:**
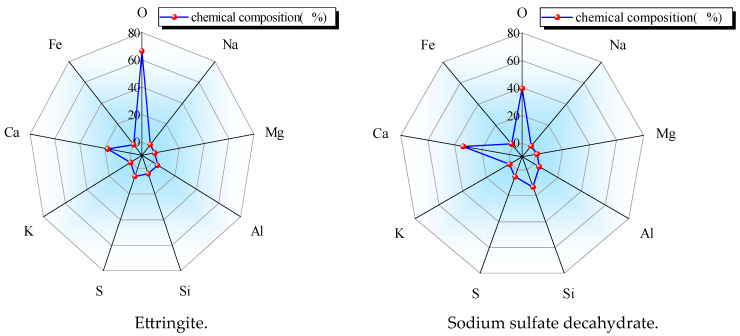
EDS results for the sample.

Sulfate attack on cement-based materials is mainly caused by the reaction of sulfate ions with calcium ions and aluminates after cement hydration. The reaction produces ettringite expansion crystals. The excess sulfate ions precipitate sodium sulfate decahydrate crystals (Na_2_SO_4_·10H_2_O) in the supersaturated state, which also causes volume expansion. The microscopic morphology of the two expansions is shown in Figure 7 and Figure 8. At measurement point 1, needle-like crystals were selected. The main elements were O (66.5%), Ca (17.6%), S (6.3%), Si (4.2%), and Al (4.2%). This crystal was composed of calcium aluminosilicate and CaSiO_3_. At measurement point 2, gel products and granular crystals were selected. The main elements were O (39.7%), Ca (33.7%), S (5.3%), Si (13.3%), Al (4.3%), Na (0.35%), Mg (1%), and Fe (2.3%). This part mainly consisted of C-S-H and ten water-saturated sodium sulfate crystals (Na_2_SO_4_·10H_2_O). The analysis suggests that under the action of sulfate erosion, the expansive erosion products of calcium aluminosilicate and ten water-saturated sodium sulfate crystals formed between the cementitious materials and the micropores led to the strength evolution of cement-stabilized macadam. The microscopic pore structure of the cement-stabilized macadam mixture determines the material’s capacity to accommodate crystalline products, so a skeleton-pore-type cement-stabilized macadam with a well-developed pore structure exhibits better sulfate erosion resistance.

**Figure 9:**
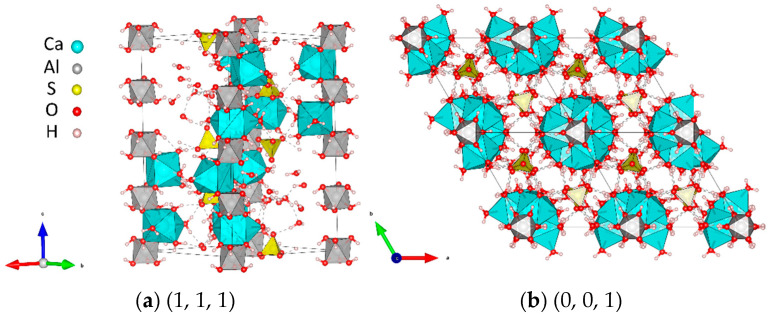
Crystal structure of ettringite.

Ettringite belongs to the trigonal system, and the unit cell parameters are c = 2.15 nm, a = b = 1.125 nm. Its crystal structure is parallel to the c-axis, so its structure is mostly prismatic under electron microscope scanning, as shown in Figure 9. Ettringite is characterized by low solubility and high strength. It combines a large amount of crystal water in its chemical structure. Its volume is about 2.5 times that of hydrated calcium aluminate, which leads the solid-phase volume to increase significantly, as shown in Equations (2)–(5).

(2) $$S{O_{4}}^{2-}+Ca^{2-}+2H_{2}O\to CaSO_{4}\cdot 3H_{2}O$$

(3) $$3CaO\cdot Al_{2}O_{3}+3\left(CaSO_{4}\cdot 3H_{2}O\right)+26H_{2}O\to 3CaO\cdot Al_{2}O_{3}\cdot 3CaSO_{4}\cdot 32H_{2}O$$

(4) $$\begin{array}{l} 4CaO\cdot Al_{2}O_{3}\cdot 13H_{2}O+3\left(CaSO_{4}\cdot 2H_{2}O\right)+14H_{2}O\\ \to 3CaO\cdot Al_{2}O_{3}\cdot 3CaSO_{4}\cdot 32H_{2}O \end{array}$$

(5) $$\begin{array}{l} 3CaO\cdot Al_{2}O_{3}\cdot CaSO_{4}\cdot 12H_{2}O+2\left(CaSO_{4}\cdot 2H_{2}O\right)+16H_{2}O\\ \to 3CaO\cdot Al_{2}O_{3}\cdot 3CaSO_{4}\cdot 32H_{2}O \end{array}$$

**Figure 10:**
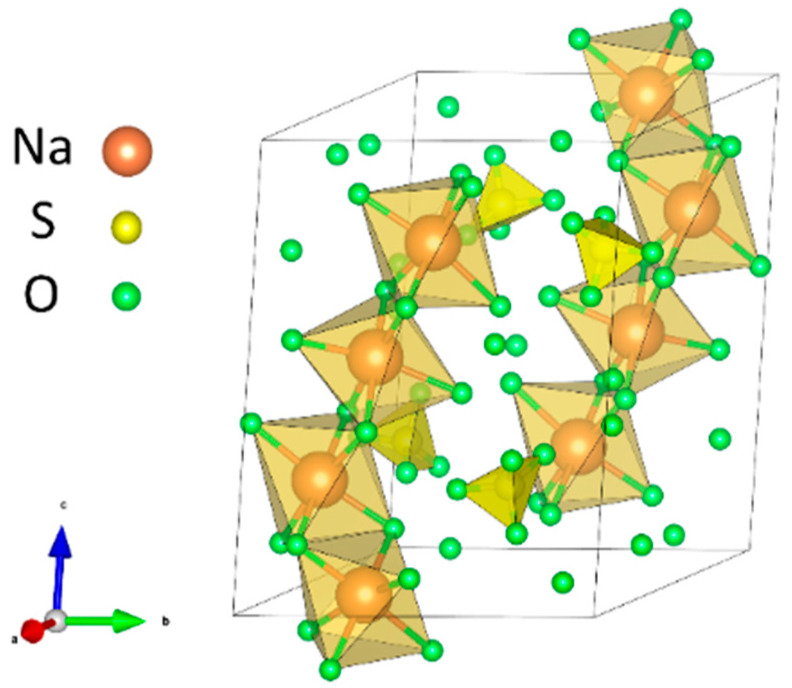
Crystal structure of sodium sulfate decahydrate.

When the concentration of sodium sulfate is high enough and the temperature is lower than 32.4 °C, the crystallization reaction of sodium sulfate salt will occur; the molecular cell diagram is shown in Figure 10. As shown in Equation (6), the reaction precipitates salts with crystal water, and the volume expansion is 3.15 times, resulting in great crystallization pressure, leading to the destruction of broken and split concrete.

(6) $$Na_{2}SO_{4}+10H_{2}O\to Na_{2}SO_{4}\cdot 10H_{2}O$$

### 3.3. Deformation and Pore Analysis in Target Gradation Specimen

**Figure 11:**
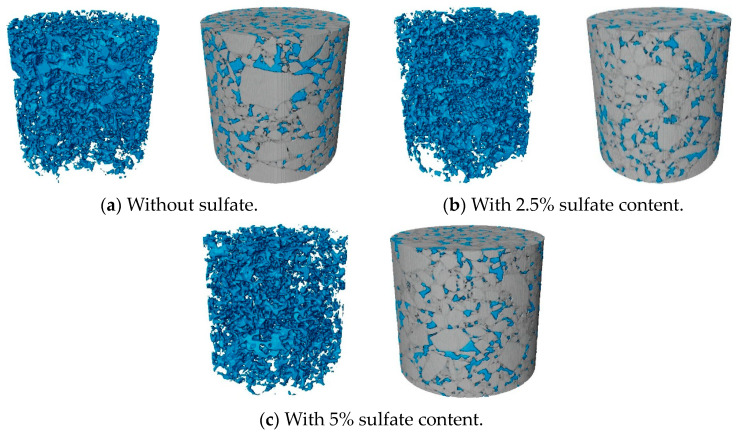
Three-dimensional reconstruction of pore structure and model.

**Figure 12:**
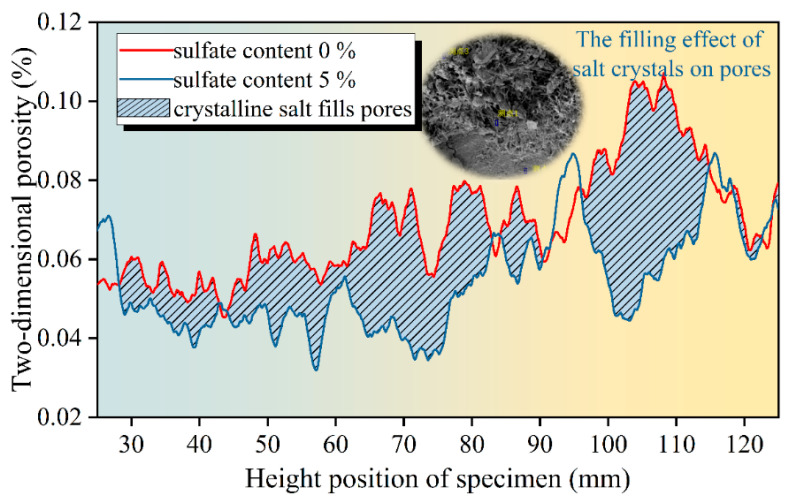
Porosity distribution curve along the height of the specimen.

According to the results of CT scanning, the three-dimensional morphology of cement-stabilized macadam specimens was reconstructed in Avizo image processing software. The image contrast was set to 0–200, and the pore domain value was set to 1–70. The pore distribution and three-dimensional morphology of the specimens under different sulfate contents after treatment are shown in Figure 11. According to the three-dimensional reconstruction model, the pore parameters of the specimen were extracted and the volume porosity was calculated. At the same time, the actual linear expansion deformation caused by the sulfate content was recorded. The results are shown in Figure 12. The linear expansion rate of the specimen is calculated as follows:

(7) $$A_{W}=\left(L_{W}-L_{0}\right)/L_{0}$$

where

$A_{W}$ is the linear expansion coefficient of the specimen under *w*% salt content;
$L_{W}$ is the height of the specimen under *w*% salt content; mm;
$L_{0}$ is the original height of specimen; mm.

Typical tomography images of the middle section of the specimen were selected, and the distribution curves of the layered porosity along the height of the specimens with sulfate contents of 0% and 5% were drawn, as shown in Figure 12. The solid line in the figure is the distribution curve of the layered porosity of different sulfate contents along the height of the specimen, and the shadowed part represents the filling effect on the pores caused by the crystals produced with 5% sulfate content. Based on the distribution of the layered porosity of the specimen along the height of the specimen, the upper porosity of the specimen is generally higher than the lower porosity of the specimen. This is because in the forming stage of the specimen and the early stage of cement hydration, the cement mortar in the specimen is wrapped by fine aggregates and deposited downward under the influence of gravity. This means that the proportion of fine aggregates in the lower part of the specimen is higher, the layered porosity is relatively low, and the porosity of the upper part of the specimen increases due to the loss of fine particles. By comparing the porosity of specimens with different sulfate contents, it can be seen that the layered porosity distribution curve of specimens with 0% sulfate content is basically higher than that of specimens with 5% sulfate content, which indicates that an increase in sulfate content will reduce the overall porosity of specimens and make the mixture denser. The layered porosity of the specimen with 5% sulfate content was taken as the baseline, and the layered porosity of the specimen with 0% sulfate content was integrated. The area of the shadowed part in the figure was calculated to be 1.50%, indicating that the expansive crystalline product produced with 5% sulfate erosion filled the 1.5% porosity of the specimen.

**Figure 13:**
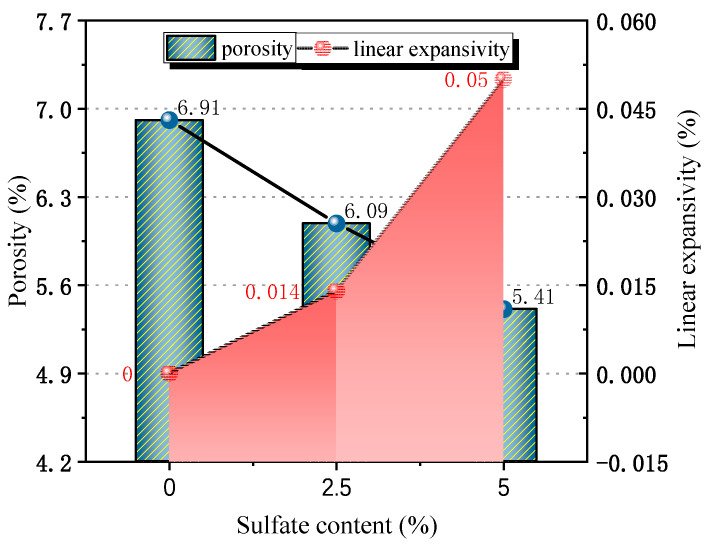
Model porosity and measured linear expansion rate.

It can be seen in Figure 13 that with an increase in sulfate content in the specimen, the overall porosity of the specimen shows a decreasing trend. When the salt content increased from 0 to 2.5%, the volume porosity decreased by 0.82%. When the sulfate content continued to increase to 5%, the volume porosity decreased by 1.5%. This shows that with the increase in sulfate content, the yield of expansive crystalline phase in cement-stabilized macadam increases, and the continuous filling of the microscopic pores of the structure leads to a decrease in overall porosity. The measured linear expansion rate of the specimen also reflects this trend. When the specimen does not contain salt, it only shows dry shrinkage deformation, so the default linear expansion rate is 0. When the sulfate content is 2.5%, the linear expansion rate of the specimen increases to 0.016%. When the sulfate content is 5%, the volume porosity increases to 0.050%. The decrease in porosity and the increase in linear expansion rate under the action of sulfate erosion indicate that the filling of microscopic pores with the crystalline phase is the main cause of expansion deformation in the specimen at the macro scale.

**Figure 14:**
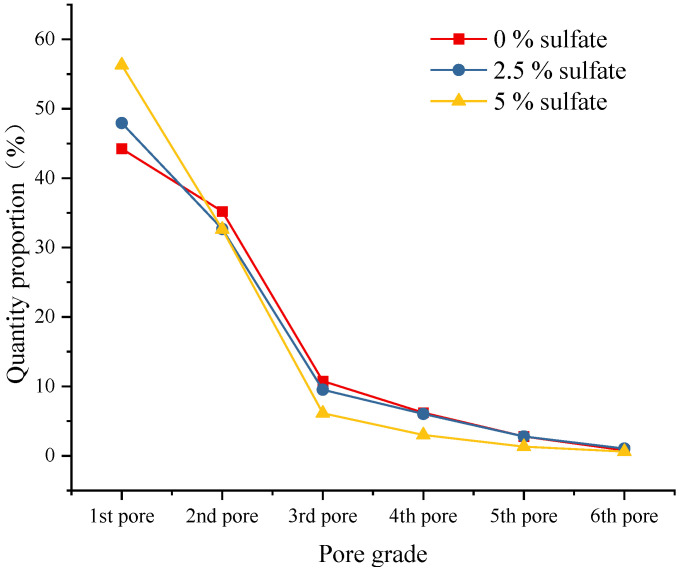
The number distribution of graded pores in the model.

**Figure 15:**
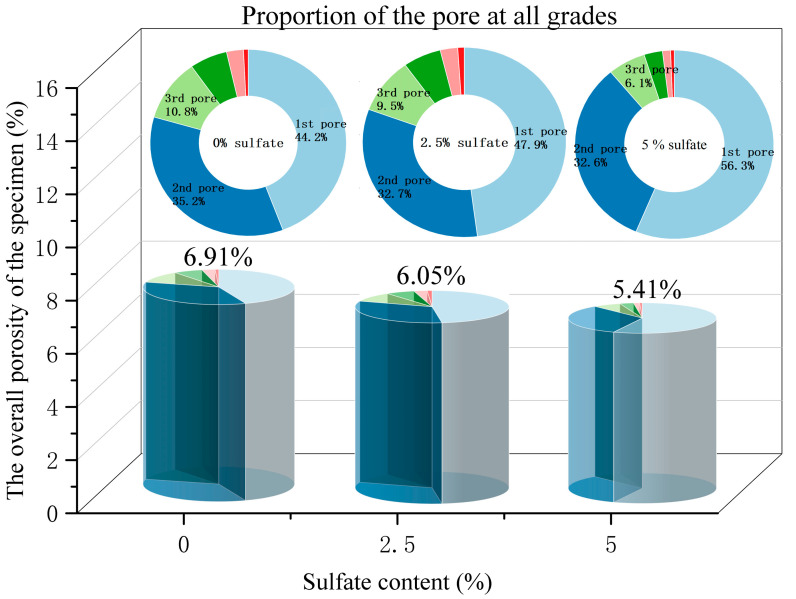
Proportion of the number of graded pores.

The pores extracted from the model are classified according to the previously described pore classification standard. The distribution of the number of pores is shown in Figure 14, and the proportion of graded pores in a single specimen is shown in Figure 15. As shown in Figure 14, the sulfate content in the specimen affects the distribution of the proportion of pores at all levels. Compared with the specimens without salt, the larger the sulfate content in the specimens, the larger the proportion of small pores, and the proportion of other pores decreases accordingly. Figure 15 shows the overall porosity of the specimen to analyze pore characteristics under sulfate attack. It can be seen that under the action of sulfate erosion, the overall porosity of the specimen decreases and the proportion of small pores increases. From the specific data, we can see that when the sulfate content increased from 0% to 5%, the overall porosity of the specimen decreased by 1.5%, and the proportion of small pores increased by 12.1%.

By analyzing the pore evolution law of the cement-stabilized macadam mixture under sulfate erosion, we found that with an increase in sulfate content, the overall porosity of the specimen decreases and the proportion of small pores increases. This suggests that the crystal is formed in the mesopores of the mixture and gradually fills and squeezes the pores, which is macroscopically manifested as salt expansion deformation.

### 3.4. Prediction of Salt Expansion Deformation in Cement-Stabilized Macadam Based on Pore Evolution

**Figure 16:**
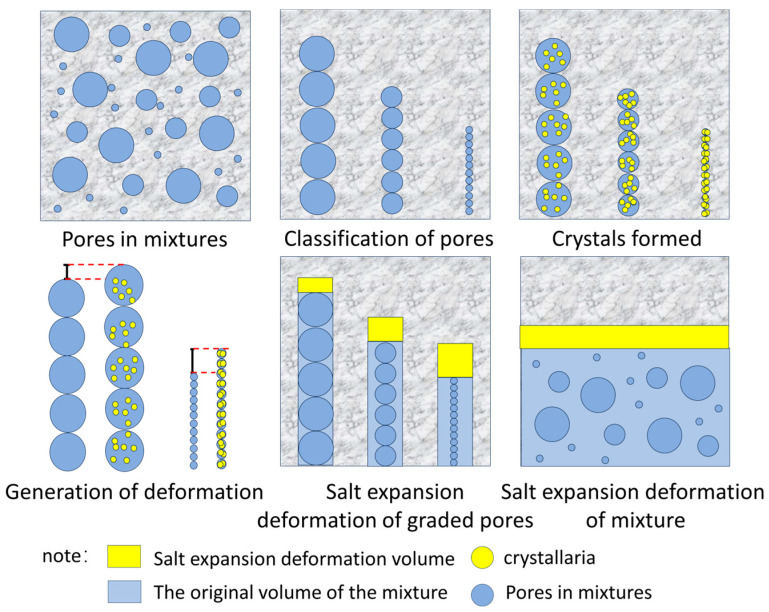
The schematic diagram of the deformation prediction model.

According to the physical expansion mechanism of sulfate erosion in cement-stabilized macadam, the mesopore structure of the mixture has a quantitative effect. In this paper, considering the different effects of pores of different sizes on salt expansion deformation under sulfate attack, macroscopic deformation is divided into the total amounts of deformation caused by pores of different sizes. Based on this, the quantitative influence of pores of different sizes on salt expansion deformation is tested via a single-grain test, and then the volume proportion of pores of different sizes in the mixture is obtained via a CT scanning test. The salt expansion deformations caused by pores of different sizes can be calculated, and, by summing them, a prediction model for the whole mixture can be obtained. The schematic diagram of this model is shown in Figure 16.

In this paper, we calculated the quantitative influence of different pores on salt expansion deformation and extracted the volume proportion of graded pores in the model. Finally, the salt expansion deformation caused by graded pores was weighted and summed, and a prediction model was obtained. The specific calculation process was as follows.

Firstly, the theoretical porosity *P_r_* and the measured linear expansion rate *ε_(w%,r)_* were obtained, and the impact factors of different grades of pores on salt expansion deformation were calculated using Equation (8).

(8) $$IF_{i_{\left(w\%,r\right)}}=\frac{\varepsilon_{\left(w\%,r\right)}}{P_{r}\times 100\%}$$

where

$\varepsilon_{\left(w\%,r\right)}$ is the linear expansion rate of the single-particle-size specimen;
$P_{r}$ is the porosity of the single-particle-size specimen, %;
$IF_{i}$ is the impact factor of the *i*-grade pore in salt expansion deformation;
w is the sulfate content,%;
r is the equivalent sphere volume radius, mm.

Then, the measured porosity *P_(w%)_* and the proportion of pores in each grade *Rn_i(w%)_* of the cement-stabilized macadam specimens under the target gradation were obtained via CT scanning. The volume ratio *Rv_i(w%)_* of each grade pore was converted using Equation (9).

(9) $$Rv_{i_{\left(w\%\right)}}=\frac{Rn_{i_{\left(w\%\right)}}\times V_{i}}{\sum V_{i}}$$

where

$Rn_{i_{\left(w\%\right)}}$ is the proportion of pore number of grade *i*, %;
$Rv_{i_{w\%}}$ is the proportion of pore volume of grade *i*, %;
$V_{i}$ is the average volume of grade *i* pores.

Finally, by calculating the linear expansion rate caused by each pore and summing them, the linear expansion rate of the specimen is obtained. The calculation formula is given as Equation (10):

(10) $$\varepsilon_{\left(w\%\right)}=\sum P_{\left(w\%\right)}\times Rv_{i_{\left(w\%\right)}}\times IF_{i_{\left(w\%,r\right)}}$$

where

$\varepsilon_{\left(w\%\right)}$ is the linear expansion rate of the target gradation specimen, %;
$P_{(w\%)}$ is the overall porosity of the specimen, %.

The impact factors of each pore with 2.5% and 5% sulfate content are calculated as shown in Table 4 and Table 5. The theoretical porosity in the table refers to the compaction porosity of a single particle size. The proportion of pore volume of each grade and the theoretical linear expansion rate of the specimen are shown in Table 6.

According to the calculation results in Table 4 and Table 5, it can be seen that the impact factors for different sizes of pores are different. The smaller the pore size, the greater the impact factor, and the structure shows greater salt expansion deformation with the same salt content. By comparing the impact factors for each grade of pores under different sulfate contents, we found that with an increase in sulfate content in the specimen, the impact factors for each grade of pores increase to varying degrees, which indicates that an increase in sulfate content will also cause the specimen to show a greater degree of salt expansion deformation.

As shown in Table 6, the theoretical calculation of the linear expansion rate of the specimen fits well with the measured value, and the error value is below 10%. This shows that the theoretical calculation method used in this paper can accurately reveal the salt expansion deformation mechanism of the specimen. It provides a reference for further analysis of the salt expansion deformation law of cement-stabilized macadam.

## 4. Discussion

The prediction model for salt expansion deformation in cement-stabilized macadam established in this paper is based on the evolution of the pore structure of mixtures caused by sulfate erosion. The model takes salt expansion deformation as the influence factor and the porosity and proportion of graded pores as the main parameters, and its main use is in predicting salt expansion deformation in cement-stabilized macadam with different sulfate contents. For each content, the porosity and the proportion of graded pores are directly obtained via CT scanning. In this paper, we mainly consider the influence of pore size and salt content on salt expansion deformation, but the salt expansion phenomenon in cement-stabilized macadam in practical engineering is also affected by temperature, salt type, humidity, and other factors, so researchers should conduct further work on the above factors.

## 5. Conclusions

The following conclusions can be drawn from the work in this paper:

(1) Considering the grading effect of different pore sizes in salt expansion deformation in cement-stabilized macadam, the influence factors of grading pores in salt expansion deformation can be calculated based on a salt expansion test on single-grain cement-stabilized macadam. The mesopore characteristics of the mixture are obtained based on a CT scan of the target-graded cement-stabilized macadam. Based on the above parameters, a prediction model for salt expansion deformation is established. It is verified that the error between the calculated value and the measured value for this model is below 10%.
(2) We calculate the influence factors in salt expansion deformation when graded pores have different salt contents. The influence factor of macropores is small, and the specimen has a strong ability to accommodate the expansion phase. The specimen shows a low level of salt expansion deformation with an increase in sulfate content. The influence factor of small pores is large, the capacity of the specimen to accommodate the expansion products is weak, and the salt expansion deformation of the specimen increases with the increase in sulfate content.
(3) It has been found that as the sulfate content increases from 0 to 5%, the porosity of cement-stabilized macadam decreases by 1.5% and the proportion of small pores increases by 12.1%. This shows that sulfate attack leads to the pores of the mixture being filled, changing the mixture’s pore structure and causing salt expansion deformation.

## Figures and Tables

**Table 1:** Single-particle-size cement-stabilized macadam salt expansion deformation test scheme.

Influencing Factor	Test Conditions				
Sodium sulfate parameter (%)	0	1.5	2.5	3.5	5
Sieve size (mm)	0.075	0.3	0.6	1.18	2.36
Theoretical aperture (mm)	0.055–0.22	0.22–0.44	0.44–0.85	0.85–1.70	1.70–3.42

**Table 2:** Pore classification standard [35].

Sieve Size (mm)	Equivalent Sphere Volume Radius (mm)	Pore Volume (μm^3^)	Pore Grade
<0.3	<0.11	<5.28 × 10^6^	1st pore
0.3–0.6	0.11–0.22	5.28 × 10^6^–4.22 × 10^7^	2nd pore
0.6–1.18	0.22–0.42	4.22 × 10^7^–3.21 × 10^8^	3rd pore
1.18–2.36	0.42–0.85	3.21 × 10^8^–2.57 × 10^9^	4th pore
2.36–4.75	0.85–1.71	2.57 × 10^9^–2.09 × 10^10^	5th pore
>4.75	>1.71	>2.09 × 10^10^	6th pore

**Table 3:** Target gradation of cement-stabilized macadam specimen.

Sieve diameter (mm)	0.075	0.3	0.6	1.2	2.4	4.8	9.5	19.0	31.5
Percentage of sieve residue (%)	1	3	6	11	18	20	62	82	100

**Table 4:** The impact factor of pores in salt expansion deformation with 2.5% sulfate content.

Sieve Size (mm)	Equivalent Sphere Volume Radius (mm)	Theoretical Porosity (%)	Measured Linear Expansion Rate (%)	Impact Factor	Pore Grade
<0.3	<0.11	30.85	1.71	0.0554	1st pore
0.3–0.6	0.11–0.22	32.36	1.02	0.0316	2nd pore
0.6–1.18	0.22–0.42	35.33	0.67	0.0189	3rd pore
1.18–2.36	0.42–0.85	37.77	0.42	0.0112	4th pore
2.36–4.75	0.85–1.71	38.87	0.27	0.0069	5th pore
>4.75	>1.71	/	/	0	6th pore

**Table 5:** The impact factor of pores in salt expansion deformation with 5% sulfate content.

Sieve Size (mm)	Equivalent Sphere Volume Radius (mm)	Theoretical Porosity (%)	Measured Linear Expansion Rate (%)	Impact Factor	Pore Grade
<0.3	<0.11	30.85	4.2	0.1361	1st pore
0.3–0.6	0.11–0.22	32.36	3.13	0.0968	2nd pore
0.6–1.18	0.22–0.42	35.33	2.27	0.0642	3rd pore
1.18–2.36	0.42–0.85	37.77	1.67	0.0441	4th pore
2.36–4.75	0.85–1.71	38.87	0.78	0.02	5th pore
>4.75	>1.71	/	/	0	6th pore

**Table 6:** The theoretical linear expansion rate of the specimen and the measured value.

Test Condition	The Proportion of Pores of Each Volume Grade (%)						Theoretical Linear Expansion Rate (%)	Measured Linear Expansion Rate (%)	Error (%)
/	1st pore	2nd pore	3rd pore	4th pore	5th pore	6th pore	/	/	/
2.5% sulfate	0.05	0.55	1.29	6.46	24.51	67.14	0.0173	0.016	8.1
5% sulfate	0.11	0.97	1.47	5.73	20.2	71.52	0.0465	0.050	−5.1

## Data Availability

The original contributions presented in this study are included in the article. Further inquiries can be directed to the corresponding author.

## References

[1] Song L., Song Z., Wang C., Wang X., Yu G. (2019). Arch expansion characteristics of highway cement-stabilized macadam base in Xinjiang, China. Constr. Build. Mater..

[2] Bao W., Tian L., Li H., Huang Z., Yin Y., Zhang Z. (2025). Study on the arch expansion characteristics of cement-stabilized macadam under the action of temperature and salt. Constr. Build. Mater..

[3] Ji F., Peng Y., Lv Q., Li W., Yu J. (2024). Pure salt expansion behavior in sulfate saline soil under negative temperature conditions. Cold Reg. Sci. Technol..

[4] Wang D., Yang X., Zhang S., Chen C., Zhao Y. (2024). Long-Term Bearing Capacity of Concrete Pile Composite Foundation under Composite Salt Erosion. Buildings.

[5] Ragoug R., Metalssi O.O., Barberon F., Torrenti J.M., Roussel N., Divet L., de Lacaillerie J.B.D.E. (2019). Durability of cement pastes exposed to external sulfate attack and leaching: Physical and chemical aspects. Cem. Concr. Res..

[6] Wang F., Yu H., Ma H., Cheng M., Guo J., Zhang J., Liu W., Gao W., Tao Q., Guo J. (2024). Splitting Tensile Mechanical Performance and Mesoscopic Failure Mechanisms of High-Performance Concrete under 10-Year Corrosion from Salt Lake Brine. Buildings.

[7] Wang C., Fang Z., Zhuang X., Chen Q., Han K., Zhou S. (2025). External sulfate attack on cement-based materials in underground structures with the stray current. Constr. Build. Mater..

[8] Wu L., Yi C., Feng Q., Huang X., Mao Z. (2024). Numerical simulation of sulfate attack in cement based materials: Considering dynamic boundary calcium concentration. Case Stud. Constr. Mater..

[9] Zou D., Qin S., Liu T., Jivkov A. (2021). Experimental and numerical study of the effects of solution concentration and temperature on concrete under external sulfate attack. Cem. Concr. Res..

[10] Chen X., Gu X., Xia X., Li X., Zhang Q. (2021). A Chemical-Transport-Mechanics Numerical Model for Concrete under Sulfate Attack. Materials.

[11] Ikumi T., Segura I. (2019). Numerical assessment of external sulfate attack in concrete structures. A review. Cem. Concr. Res..

[12] Yin G.J., Wen X.D., Miao L., Cui D., Zuo X.B., Tang Y.J. (2023). A Review on the Transport-Chemo-Mechanical Behavior in Concrete under External Sulfate Attack. Coatings.

[13] Yin G.J., Shan Z.Q., Miao L., Tang Y.J., Zuo X.B., Wen X.D. (2022). Finite element analysis on the diffusion-reaction-damage behavior in concrete subjected to sodium sulfate attack. Eng. Fail. Anal..

[14] Shao W., Li Q., Zhang W., Shi D., Li H. (2023). Numerical modeling of chloride diffusion in cement-based materials considering calcium leaching and external sulfate attack. Constr. Build. Mater..

[15] Xiao Q.H., Cao Z.Y., Guan X., Li Q., Liu X.L. (2019). Damage to recycled concrete with different aggregate substitution rates from the coupled action of freeze-thaw cycles and sulfate attack. Constr. Build. Mater..

[16] Tang K., Mao X.S., Wu Q., Zhang J.X., Huang W.J. (2020). Influence of Temperature and Sodium Sulfate Content on the Compaction Characteristics of Cement-Stabilized Macadam Base Materials. Materials.

[17] Li T.F., Xiao X.P., Yan R.H., Xie K., Li J.S., Dai R.H. (2024). Research on deterioration mechanism of graded gravel in high-speed railway subgrade layer based on machine vision. Case Stud. Constr. Mater..

[18] Zhang Y., Zuo L., Yang J., Cai X.N., Zhao Y., Zeng X. (2019). Effect of cementitious capillary crystalline waterproofing coating on the gas permeability of mortar. Struct. Concr..

[19] Xue W., Zhou C., Zhang W., Mao Y., Peng X. (2025). Hydro-mechanical coupling characteristics and mechanism of salt intrusion freeze-thaw concrete under complex stress paths. J. Build. Eng..

[20] Wang Q., Liu J., Li X., Wang P., Liu J., Sun M. (2025). Expansion mechanism of sulfate attack on cement-treated aggregates under freeze–thaw cycles. J. Zhejiang Univ.-Sci. A.

[21] Liu Z., Zhang F., Deng D., Xie Y., Long G., Tang X. (2017). Physical sulfate attack on concrete lining—A field case analysis. Case Stud. Constr. Mater..

[22] Wang B., Li G., Panesar D.K. (2021). A study of variables that affect the process of sulfate attack of cement--based materials subjected to stray current. Struct. Concr..

[23] Yin G.J., Lang Y.J., Miao L., Wen X.D., Shao J.J., Zuo X.B. (2023). Numerical simulation on the crystallization-induced mechanical response in hardened cement paste under external sulfate attack. Ocean. Eng..

[24] Wu T., Jin L., Fan T., Qiao L., Liu P., Zhou P., Zhang Y. (2023). A multi-phase numerical simulation method for the changing process of expansion products on concrete under sulfate attack. Case Stud. Constr. Mater..

[25] Shan Z.Q., Yin G.J., Wen X.D., Miao L., Wang S.S., Zuo X.B. (2024). Numerical simulation on transport-crystallization-mechanical behavior in concrete structure under external sulfate attack and wetting–drying cycles. Mater. Des..

[26] Zhou H., Ouyang W., Zou S., Xu S. (2024). The Control of the Expansion or Compression of Colloidal Crystals Lattice with Salt Solution. Nanomaterials.

[27] Zhang M., Lv H., Zhou S., Wu Y., Zheng X., Yan Q. (2023). Study on the Frost Resistance of Composite Limestone Powder Concrete against Coupling Effects of Sulfate Freeze–Thaw. Buildings.

[28] Wang C., Chen Y., An B., Guo Q., Wang Y. (2024). Impact of salt erosion on mechanical and drying shrinkage performance of cement stabilized macadam. Front. Mater..

[29] Sun D., Cao Z., Huang C., Wu K., De Schutter G., Zhang L. (2022). Degradation of concrete in marine environment under coupled chloride and sulfate attack: A numerical and experimental study. Case Stud. Constr. Mater..

[30] Li Y., Wang J., Yang Y., Tang T. (2024). Tensile Performance and Aging Increase Factor Constitutive Model of High-Strength Engineered Cementitious Composites under Sulfate Salt Attack. Buildings.

[31] Wang J., Niu D. (2016). Influence of freeze–thaw cycles and sulfate corrosion resistance on shotcrete with and without steel fiber. Constr. Build. Mater..

[32] Wang Q., Kunther W., Li Y., Visalakshi T., Gomasa R., Amroun S., De Souza D.J., Kasaniya M., Tole I., Li X. (2025). Sulfate attack testing approaches from concrete to cement paste: A review by RILEM TC 298-EBD. Mater. Struct..

[33] Wu Q., Ma Q., Huang X. (2021). Mechanical properties and damage evolution of concrete materials considering sulfate attack. Materials.

[34] Siad H., Kamali-Bernard S., Mesbah H.A., Escadeillas G., Mouli M., Khelafi H. (2013). Characterization of the degradation of self-compacting concretes in sodium sulfate environment: Influence of different mineral admixtures. Constr. Build. Mater..

[35] (2019). Technical Specification for Construction of Cement Stabilized Macadam Base.

[36] Chen Z., Yuan W., Gao C. (2006). Research on Design Method of Multilevel Dense Built-in Gradation. China Journal of Highway and Transport.

